# Cloning-independent markerless gene editing in *Streptococcus sanguinis*: novel insights in type IV pilus biology

**DOI:** 10.1093/nar/gkw1177

**Published:** 2016-11-28

**Authors:** Ishwori Gurung, Jamie-Lee Berry, Alexander M. J. Hall, Vladimir Pelicic

**Affiliations:** MRC Centre for Molecular Bacteriology and Infection, Imperial College London, London, UK

## Abstract

*Streptococcus sanguinis*, a naturally competent opportunistic human pathogen, is a Gram-positive workhorse for genomics. It has recently emerged as a model for the study of type IV pili (Tfp)—exceptionally widespread and important prokaryotic filaments. To enhance genetic manipulation of *Streptococcus sanguinis*, we have developed a cloning-independent methodology, which uses a counterselectable marker and allows sophisticated markerless gene editing *in situ*. We illustrate the utility of this methodology by answering several questions regarding Tfp biology by (i) deleting single or mutiple genes, (ii) altering specific bases in genes of interest, and (iii) engineering genes to encode proteins with appended affinity tags. We show that (i) the last six genes in the *pil* locus harbouring all the genes dedicated to Tfp biology play no role in piliation or Tfp-mediated motility, (ii) two highly conserved Asp residues are crucial for enzymatic activity of the prepilin peptidase PilD and (iii) that pilin subunits with a C-terminally appended hexa-histidine (6His) tag are still assembled into functional Tfp. The methodology for genetic manipulation we describe here should be broadly applicable.

## INTRODUCTION

The Gram-positive bacterium *Streptococcus sanguinis* is a normal inhabitant of the human mouth where it is found in dental plaque (1). Although its presence is beneficial for dental health because it interferes with the colonisation by caries-causing *Streptococcus mutans* (1), *S. sanguinis* is also an important opportunistic pathogen being one of the most common causative agents of infective endocarditis (2). Due to its rapid growth, relatively small genome (3), and natural competence for DNA transformation (4), *S. sanguinis* is a genetic workhorse. Indeed, allelic exchange mutagenesis is rapidly and easily achieved by directly transforming *S. sanguinis* with PCR products in which the upstream (Up) and downstrean (Dn) regions of a target gene are spliced to an antibiotic resistance cassette. This has facilitated the design of a comprehensive and ordered collection of mutants, one of the few available in bacteria (5).

Recently (6), *S. sanguinis* has emerged as a new model for the study of Tfp—long surface-exposed filaments which are the paradigm of a large group of evolutionary related filamentous nano-machines known as type IV filaments (Tff) (7). Tff, which mediate an astonishing array of functions ranging from surface-associated motility to electrical conductance (8), have been intensively studied for decades because they are almost universal in Bacteria and Archaea. Tff are composed of pilin subunits with a characteristic N-terminal motif (a hydrophilic leader peptide followed by a tract of 21 predominantly hydrophobic residues) (9). Upon cleavage of their leader peptide by a dedicated aspartic acid protease—the prepilin peptidase (10)—pilins are polymerised in filaments by complex and conserved multi-protein membrane-embedded machineries (7). The molecular mechanisms of both Tff assembly and the diverse functions these filaments mediate remain poorly understood. *S. sanguinis* is an attractive model to study Tff biology because we have recently shown that it assembles retractable Tfp using a rudimentary machinery (6), more basic than in Gram-negative species. *S. sanguinis* pilus retraction, which is under the dependence of the PilT ATPase (6), generates huge tensile forces—like in Gram-negative bacteria (11,12)—and promotes intense surface-associated motility, which is readily seen as spreading zones around colonies on plates. The peculiarity of *S. sanguinis* Tfp is that they are are composed of two pilins (PilE1 and PilE2) in comparable quantities (5), rather than one as normally seen.

Methods for sophisticated gene editing in *S. sanguinis* are lacking, which prevents us from harnessing the full potential of this species as a model for genomics and/or Tfp biology. In particular, there is no easy method for creating markerless mutations, although a couple of unmarked deletion mutants have been constructed by transforming *S. sanguinis* in the absence of selection and identifying mutants by extensive PCR screening (13,14). Markerless mutations are typically engineered using counterselectable markers (15). Of the many counterselectable markers that have been described in the literature, only few are host-genotype-independent and work in wild-type (WT) isolates. Among these, the *pheS* gene encoding the α subunit of the phenylalanyl-tRNA synthetase, which specifically catalyses the attachment of Phe to its cognate tRNA, is of particular interest because it is universally conserved. It has been shown, originally in *Escherichia coli* (16), that a mutant PheS* protein containing a single Ala substitution in a conserved Gly residue displays relaxed substrate specificity and becomes capable of aminoacylating phenylalanine analogs such as *p*-chloro-phenylalanine (*p*-Cl-Phe). Consequently, bacteria carryng a mutant pheS* allele become sensitive to *p*-Cl-Phe (16), presumably because of the incorporation of this analog into proteins. This has been confirmed in species as distant as Firmicutes, and pheS* has therefore been used as a counterselectable marker to engineer markerless gene deletion mutants in a wide range of bacteria (16–18).

In order to further cement *S. sanguinis* as a Gram-positive workhorse for genetics/genomics and Tfp biology, we aimed to adapt the pheS* counterselectable marker for use in this species. As reported here, we designed a cloning-independent gene editing methodology allowing not only the creation of markerless gene deletion mutants as reported in other species, but also the engineering of strains expressing proteins with specific amino acid substitutions or appended affinity tags.

## MATERIALS AND METHODS

### Strains and growth conditions

The sequenced *S. sanguinis* 2908 (6) strain was used in this study. Bacteria were grown on plates containing Todd Hewitt (TH) medium (Difco) and 1% agar (Difco). Plates were incubated overnight (O/N) at 37°C in 3.5 l anaerobic jars (Oxoid), under anaerobic conditions generated using Anaerogen sachets (Oxoid). Liquid cultures were grown statically under aerobic conditions in THT, *i.e*. TH broth containing 0.05% tween 80 (Merck) to limit bacterial clumping. 500 μg/ml kanamycin (Sigma) was used for antibiotic selection. 15 mM *p*-Cl-Phe (Sigma), was used for counterselection. The compound (a mixture of L- and D-isomers) was added to culture media prior to autoclaving.

*Streptococcus sanguinis* genomic DNA was prepared from O/N liquid cultures using the kit XIT Genomic DNA from Gram-Positive Bacteria (G-Biosciences), as instructed by the manufacturer. All PCR and splicing PCR (sPCR) were done using high-fidelity Herculase II Fusion DNA Polymerase (Agilent), as described elsewhere (6). Primers that were used are listed in Table 1. PCR products were routinely sequenced by Sanger sequencing and analysed by agarose gel electrophoresis.

**Table 1.** Primers used in this study

Name	Sequence^a^	Used for
*pheS*-F	ATGACGAAAACGATTGAAGAAC	amplifying *pheS*
*pheS*-R	AGCCATtgataatatctcctCTACTTAAACTGCTGAGAAAAAC	amplifying *pheS*
*pheS*-A316G-1	GAGAAGTACTCTGGATTTG**G**CTTTGGTCTCGGCCAAGAG	site-directed mutagenesis of *pheS*
*pheS*-A316G-2	CTCTTGGCCGAGACCAAAG**C**CAAATCCAGAGTACTTCTC	site-directed mutagenesis of *pheS*
*aph*F	AAGTAGaggagatattatcaATGGCTAAAATGAGAATATCACC	amplifying *aphA-3*
*aph*R	CTAAAACAATTCATCCAGTAAAA	amplifying *aphA-3*
*pilT*-F1	CCTCAGGATGGACGGATTGA	amplifying Up *pilT*
*pilT*-R1	GTTCTTCAATCGTTTTCGTCATCCTAAACTTCCCCTTCTAGACT	amplifying Up *pilT*
*pilT*-F2	TTTTACTGGATGAATTGTTTTAGAAGTCGAAAAAGCCCTAGGAA	amplifying Dn *pilT*
*pilT*-R2	TCAAACATTGGCAGCATGACA	amplifying Dn *pilT*
*pilT*-R3	TTCCTAGGGCTTTTTCGACTTCCTAAACTTCCCCTTCTAGACT	splicing Up and Dn *pilT*
*pilT*-F3	AGTCTAGAAGGGGAAGTTTAGGAAGTCGAAAAAGCCCTAGGAA	splicing Up and Dn *pilT*
2229-F1	TGCCAAAGGTCGGTCTATGT	amplifying Up SSV_2229
2229-R1	GTTCTTCAATCGTTTTCGTCATTTTTTCTATCCATTTCTATTGTCGCTT	amplifying Up SSV_2229
2224-F2	TTTTACTGGATGAATTGTTTTAGCTTTGAACTCAGACAGAAAGGGG	amplifying Dn SSV_2224
2224-R2	TACACATGATCCCCAGCCAG	amplifying Dn SSV_2224
2229-R3	CTTTCTGTCTGAGTTCAAAGTTTTTCTATCCATTTCTATTGTCGCTT	splicing Up 2229 and Dn 2224
2224-F3	AATAGAAATGGATAGAAAAACTTTGAACTCAGACAGAAAGGGG	splicing Up 2229 and Dn 2224
*pilD*-F1	CCGTTTTTCGATACCAAGGA	amplifying Up *pilD*
*pilD*-R1	GTTCTTCAATCGTTTTCGTCATAATTTTTCCCTTTTTATACTC	amplifying Up *pilD*
*pilD*-F2	TTTTACTGGATGAATTGTTTTAGGAGTATCATGGCGGTCATCC	amplifying Dn *pilD*
*pilD*-R2	TTTAGAGCCCCAAAGAGCAA	amplifying Dn *pilD*
*pilD*-F	GGGAAGTCTAAGTTTGACACCG	cloning *pilD* in pCR8/GW/TOPO
*pilD*-R	CCATTTCTATTGTCGCTTTTGGT	cloning *pilD* in pCR8/GW/TOPO
*pilD*-D116A-1	GTCTCATTATCGGCTATATCG**C**TTTTGATACTCAGTACATCTC	site-directed mutagenesis of *pilD*
*pilD*-D116A-2	GAGATGTACTGAGTATCAAAA**G**CGATATAGCCGATAATGAGAC	site-directed mutagenesis of *pilD*
*pilD*-D179A-1	GCTTTCGGGATGGGAG**C**CATTCTCTACTTAGC	site-directed mutagenesis of *pilD*
*pilD*-D179A-2	GCTAAGTAGAGAATG**G**CTCCCATCCCGAAAGC	site-directed mutagenesis of *pilD*
*PilD*-R3	TTCCCTTTTTATACTCTTTGAAAGTCTC	splicing homology arms *pilD*
PilD-F3	GAGTATCATGGCGGTCATCC	splicing homology arms *pilD*
*pilE1*-F1	CAGGCCGGTGAAAAGACTG	amplifying Up *pilE1*
*pilE1*-R1	GTTCTTCAATCGTTTTCGTCATTTTGAATAGATCTCCTGTTTTT	amplifying Up *pilE1*
*pilE1*-F2	TTTTACTGGATGAATTGTTTTAGCGACTGGTCTGCTAATGGTG	amplifying Dn *pilE1*
*pilE1*-R2	GCTCTGTTGAAGGATCCACG	amplifying Dn *pilE1*
*pilE1*-R3	TTAGTGATGGTGATGGTGATGGTTTGAGTTTACACCATTAGCAG	appending a 6His tag to *pilE1*
*pilE1*-F3	CATCACCATCACCATCACTAATTGTCAAATCATCTAAATAAGATGTA	appending a 6His tag to *pilE1*

^a^Lower case is used for the RBS region between *pheS* and *aphA-3*. Regions of complementarity for sPCR are underlined. Mismatched bases generating mutations are in bold.

The pheS*aphA-3 double cassette was constructed as follows. The *pheS* gene from 2908 genome was amplified from start to stop codon using *pheS*-F and *pheS*-R primers (Table 1). The *pheS*-R primer contains an overhang immediately after the stop codon of *pheS*, corresponding to a canonical ribosomal binding site (RBS) (AGGAGA) followed by 8 bases before the first two codons of *aphA-3*. This PCR product was cloned directly in pCR8/GW/TOPO (Invitrogen), which was used as a template to generate a pheS* mutant allele with complementary *pheS-*A316G-1/*pheS-*A316G-2 primers (Table 1) and the Quickchange site-directed mutagenesis kit (Stratagene) as previously described (19). The mutant pheS* allele was amplified with *pheS*-F and *pheS*-R primers and spliced to a promoterless *aphA-3* gene amplified with *aph*F and *aph*R primers (Table 1). The *pheS*-R and *aph*R primers contained 26-mer regions of complementarity. The spliced PCR product was cloned directly in pCR8/GW/TOPO and the double pheS*aphA-3 double cassette was verified by sequencing.

All primary mutants were constructed as previously described (6). In brief, for each target gene, 700–1200 bp Up and Dn products were amplified using suitable F1/R1 and F2/R2 pairs of primers (Table 1). R1 and F2 were designed to cleanly delete target genes from their start codon to ∼30 bp before their stop codon. In addition, R1 and F2 primers contained 22-mer and 23-mer overhangs complementary to the *pheS*-F and *aph*R primers used to amplify the 1859 bp promoterless pheS*aphA-3 double cassette. Equal volumes of the three PCR products (Up, Dn and pheS*aphA-3) were combined and spliced together by PCR. Spliced PCR products (5 μl) were directly transformed into strain 2908 (see below) and allelic exchange mutants were selected by plating on kanamycin-containing plates and growing O/N under anaerobic conditions. Genomic DNA was extracted from at least two Km^R^ transformants and it was tested by PCR and sequenced using the corresponding F1 and R2 primers, in order to confirm that the desired allelic exchange had taken place.

Secondary mutants were constructed by directly transforming the following sPCR products into suitable primary mutants and plating transformants on plates containing 15 mM *p*-Cl-Phe. Markerless allelic exchange mutants, which are Km^S^, were identified by re-streaking *p*-Cl-Phe^R^ transformants on plates with and without kanamycin. The markerless *ΔpilT* in-frame deletion mutant was constructed by transforming the ΔpilT::pheS*aphA-3 primary mutant with a sPCR product in which the Up and Dn regions of *pilT* amplified respectively with *pilT*-F1/*pilT*-R3 and *pilT*-F3/*pilT*-R2 (Table 1) were directly spliced together. Similarly, the markerless *Δ*2229-2224 in-frame deletion mutant was constructed by transforming the *Δ*2229-2224::pheS*aphA-3 primary mutant with a sPCR product in which the Up and Dn regions of SSV_2229 and SSV_2224 amplified respectively with 2229-F1/2229-R3 and 2224-F3/2224-R2 (Table 1) were spliced together. To construct the missense mutants encoding PilD_D116A_ and PilD_D179A_ variants, we first amplified the *pilD* gene from 2908 genome using *pilD*-F and *pilD*-R primers (Table 1) and cloned it directly in pCR8/GW/TOPO. This plasmid was used as a template to generate the desired mutant *pilD* alleles using the Quickchange site-directed mutagenesis kit, and suitable complementary primers *pilD*-D116A-1/*pilD*-D116A-2 or *pilD*-D179A-1/*pilD*-D179A-2 (Table 1). The mutant *pilD* alleles were then amplified from these resulting plasmids with *pilD*-F/*pilD*-R and spliced to Up and Dn regions of *pilD* amplified with *pilD*-F1/*pilD*-R3 and *pilD*-F3/*pilD*-R2 (Table 1), and transformed in a ΔpilD::pheS*aphA-3 primary mutant. Finally, we used *pilE1*-F1/*pilE1*-R3 and *pilE1*-F3/*pilE1*-R2 primers (Table 1) to construct a *pilE1_6His_* by sPCR, which was transformed in the ΔpilE1::pheS*aphA-3 primary mutant.

### Transformation of *S. sanguinis*

Strain 2908 was transformed as described elsewhere (6), with minor modifications. In brief, bacteria grown O/N in THTS–THT supplemented with 2.5% heat-inactivated horse serum (Sigma)–were back-diluted 1/500 in THTS and incubated at 37°C for 1–1.5 h. Competence was then induced using 212 ng/ml synthetic competence stimulating peptide (20) before 100 ng of transforming DNA was added. After incubation for 1–1.5 h at 37°C, the mixture was plated on suitable agar plates, which were grown O/N under anaerobic conditions.

### Immunoblotting

*Streptococcus sanguinis* whole-cell protein extracts were prepared using a FastPrep-24 homogeniser (MP Biomedicals) and quantified as described elsewhere (6). Separation of the proteins by SDS-PAGE, subsequent blotting to Amersham Hybond ECL membranes (GE Healthcare) and blocking were carried out using standard molecular biology techniques. To detect PilE1 and PilE2, we used previously described (6) primary rabbit antibodies (1/2000 dilution), an ECL HRP-linked anti-rabbit antibody (GE Healthcare) (1/10 000 dilution), and Amersham ECL Prime (GE Healthcare). To detect His-tagged proteins, we used a commercial HRP-linked anti-6His antibody (Sigma) at 1/10 000 dilution.

### Tfp purification and detection

*Streptococcus sanguinis* 2908 Tfp were purified as described elsewhere (6). Liquid cultures were grown O/N in THT. The next day, these cultures were used to re-inoculate 100 ml of THT and grown statically until the OD_600_ reached 1–1.5, at which point OD were normalized if necessary. Bacteria were pelleted at 4°C by centrifugation for 10 min at 6000 *g* and pellets were re-suspended in 2 ml resupension buffer (20 mM Tris pH7.5, 50 mM NaCl). This suspension was vortexed for 2 min at full speed to shear Tfp, before bacteria were pelleted again as above. Supernatant (1.8 ml) was then transferred to a new tube and the pili were pelleted at 4°C by ultra-centrifugation for 1 h at 100 000 *g*. Pellets were resuspended in 80 μl resupension buffer, separated by SDS-PAGE and gels were stained using Bio-Safe Coomassie (Bio-Rad). Purified filaments were visualised by transmission electron microscopy (TEM) after negative staining as described elsewhere (6).

### Twitching motility assays

Twitching motility was assessed macroscopically on agar plates as described elsewhere (6). In brief, bacteria grown O/N were streaked as straight lines on freshly poured TH plates containing 1% agar (Eiken Chemicals), which were incubated several days in a jar in the presence of water to ensure high humidity.

## RESULTS

### Design of a double cassette allowing positive and negative selection in *S. sanguinis*, and cloning-independent markerless gene deletion

**Figure 1.**
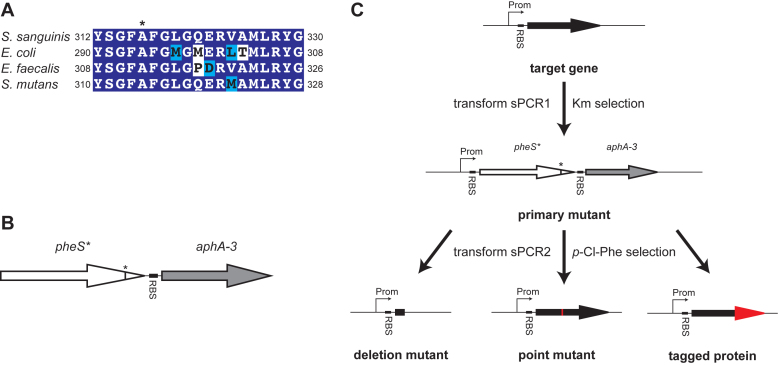
Cloning-independent strategy for markerless gene editing in *S. sanguinis*. (**A**) Multiple sequence alignment of the C-terminal region of PheS encompassing the conserved Ala residue (indicated by *) in *S. sanguinis, E. coli, E. faecalis* and *S. mutans*. Residues were shaded in dark blue (when identical), light blue (when conserved) or unshaded (when different). (**B**) Design of a promoterless double cassette for positive and negative selection. This double cassette consists of the S. sanguinis pheS* mutant gene, which contains a point mutation in codon 316 and encodes a mutant PheS_A316G_ protein expected to confer sensitivity to *p*-Cl-Phe. This double cassette also contains the *aphA-3* gene, which encodes an aminoglycoside phosphotransferase conferring resistance to kanamycin (21). The two genes are separated only by 14 bp, encompassing a RBS in front of *aphA-3*, and are expected to be co-transcribed. (**C**) Use of the double cassette for markerless gene editing in *S. sanguinis*. In the first step, the Up (up to the start codon) and Dn regions of the target gene are amplified by PCR, and spliced with the double cassette, which puts the promoterless pheS*aphA-3 under the control of the promoter (Prom) of the target gene. The sPCR1 product is directly transformed in *S. sanguinis*, and primary mutants, in which the target gene is cleanly replaced by the double cassette, are selected on plates containing kanamycin. One primary mutant is then directly transformed by sPCR2 products in which the target gene has either been deleted, engineered to contain a point mutation, or fused to a sequence encoding an affinity tag. The markerless mutants in which the double cassette has been replaced by the engineered mutation are highly enriched by plating on *p*-Cl-Phe because they are expected to be *p*-Cl-Phe^R^.

In several phylogenetically distant species, a missense Ala mutation in a conserved Gly residue in PheS renders bacteria susceptible to the phenylalanine analog *p*-Cl-Phe (16–18). Sequence alignements show that this Ala residue is conserved in *S. sanguinis* PheS (Figure 1A), suggesting that a PheS_A316G_ mutant could also result in sensitivity to *p*-Cl-Phe in this species. We therefore amplified the *pheS* gene from the sequenced *S. sanguinis* 2908 strain (6) and engineered a point mutation in codon 316 by site-directed mutagenesis, yielding a mutant pheS* gene encoding a PheS_A316G_ variant. We then used sPCR to join pheS* and *aphA-3* that confers resistance to kanamycin (21), thereby designing a double cassette for positive and negative selection in *S. sanguinis* (Figure 1B). The two genes are combined into one synthetic promoterless operon so that their expression is driven by the promoter of the gene which is being targeted.

The first step of our two-step gene editing strategy involves the creation of a primary mutant in which the target gene is replaced by the double cassette (Figure 1C). Creation of that primary mutant involves no cloning steps since a sPCR1 reaction, which splices the double cassette with the Up and Dn regions of the target gene, is transformed directly into *S. sanguinis* and selected on kanamycin. The Dn product usually contains the last ∼30 bp of the target gene, ensuring preservation of RBS that might be used by the gene immediately downstream, which is important for operonic structures. In the second step of our two-step gene editing strategy, the primary mutant is directly transformed by a sPCR2 product in which the target gene has either been deleted, engineered to contain a point mutation, or fused to a sequence encoding an affinity tag (Figure 1C). The desired markerless mutants are expected to be highly enriched by plating transformants on *p*-Cl-Phe plates, which counterselects most of the clones still containing the double cassette.

**Figure 2.**
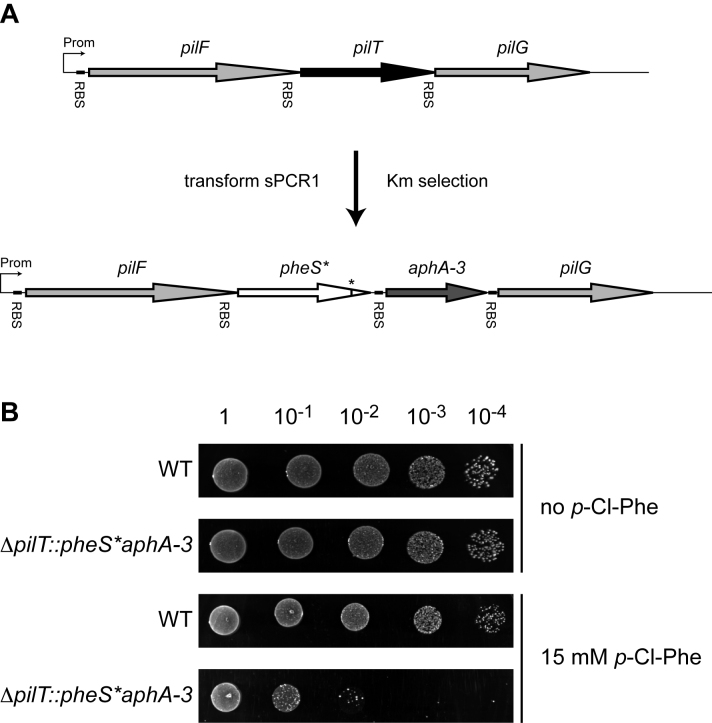
The pheS*aphA-3 double cassette allows positive and negative selection in *S. sanguinis*. (**A**) Positive selection. Using sPCR, the pheS*aphA-3 cassette was spliced to the upstream and downstream regions of the *pilT* gene, which encodes the motor powering retraction of Tfp. The sPCR reaction was directly transformed in *S. sanguinis* 2908 and ΔpilT::pheS*aphA-3 primary mutants, in which the target *pilT* gene is cleanly replaced by the double cassette, were readily selected on plates containing kanamycin. (**B**) Negative selection. Overnight liquid cultures of the WT strain and ΔpilT::pheS*aphA-3 primary mutant were adjusted at the same OD_600_ before serial 10-fold dilutions were spotted on plates with and without *p*-Cl-Phe. The plates were incubated at 37°C and photographed the next day.

**Figure 3.**
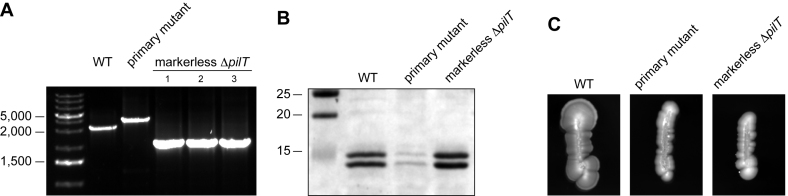
Creation of in-frame gene deletion mutants. A markerless *ΔpilT* deletion mutant is piliated and non-motile. (**A**) PCR analysis of Km^S^ clones selected on *p*-Cl-Phe plates after transformation of the ΔpilT::pheS*aphA-3 primary mutant by a sPCR2 product in which the Up and Down regions flanking the *pilT* gene have been spliced together. WT and primary mutant have been included as controls. The expected amplicons are 3,198 bp for the WT, 4,027 bp for the primary mutant, and 2,168 bp for markerless *ΔpilT* deletion mutants. Sizes shown are in bp. (**B**) Analysis of piliation. Tfp purifed by shearing/ultra-centrifugation were separated by SDS-PAGE and stained with Coomassie blue. WT and ΔpilT::pheS*aphA-3 primary mutant were included as controls. Samples were prepared from cultures adjusted to the same OD_600_, and identical volumes were loaded in each lane. A molecular weight marker was run in the first lane. Molecular weights in kDa are indicated. (**C**) Analysis of motility. Bacteria were streaked on TH plates, incubated several days at 37°C in a humid atmosphere before the plates were photographed.

As a proof of principle, we targeted the *pilT* gene encoding the molecular motor powering pilus retraction and twitching motility (Figure 2A) (6). We first spliced our double cassette with the Up and Dn regions of *pilT*, transformed the resulting sPCR1 product directly into 2908 and selected on kanamycin. Hundreds of Km^R^ transformants were readily obtained, showing that *aphA-3* was efficiently expressed from the promoter of the *pilFTG* operon (Figure 2A). Critically, the ΔpilT::pheS*aphA-3 primary mutant exhibited a dramatic sensitivity to *p*-Cl-Phe, while the WT strain was unaffected (Figure 2B). This confirmed that PheS_A316G_ can be used as a counterselectable marker in *S. sanguinis*, and that our pheS*aphA-3 double cassette allows both positive and negative selections in this species. We next transformed the ΔpilT::pheS*aphA-3 primary mutant with a sPCR2 product in which the Up and Dn of *pilT* were spliced together to generate an in-frame markerless deletion (Figure 1C), and plated on *p*-Cl-Phe plates. Replica plating of *p*-Cl-Phe^R^ clones showed that a majority of them were Km^S^ (Table 2), suggesting that they indeed corresponded to in-frame *ΔpilT* deletion mutants. This was confirmed by PCR (Figure 3A) and sequencing for a few clones. The markerless *ΔpilT* mutant had the same phenotype as a previously constructed marked mutant (6). Filaments purified from the markerless *ΔpilT* mutant and analysed by Coomassie staining after SDS-PAGE (Figure 3B) were indistinguishable from those purified from the WT strain, *i.e*. composed of the two major pilins PilE1 and PilE2 (6). These filaments were not functional and could not mediate twitching motility, as revealed by the absence of the thin spreading zones seen around WT bacteria grown on agar plates (Figure 3C) (6). This is an important result that confirms that the unmarked deletion that we introduced in *pilT* has no polar effect on the downstream *pilG* gene, which is essential for Tfp biogenesis (6) and is likely to be co-transcribed with *pilT* (Figure 2A). Therefore, our technology is applicable for in-frame markerless gene deletion in *S. sanguinis*, even for genes in complex operonic structures.

**Table 2.** Frequencies of desired mutants obtained in the various experiments

Markerless mutant	*n*	% mutant ± standard deviation
*ΔpilT*	6	66.4 ± 10.8
*Δ*2229-2224	8	45.3 ± 18.2
PilD_D116A_	8	45.3 ± 20.0
PilD_D179A_	8	47.8 ± 22.8

### Deletion of the last six genes in *S. sanguinis pil* locus shows that they are dispensable *en bloc* for piliation and twitching motility

**Figure 4.**
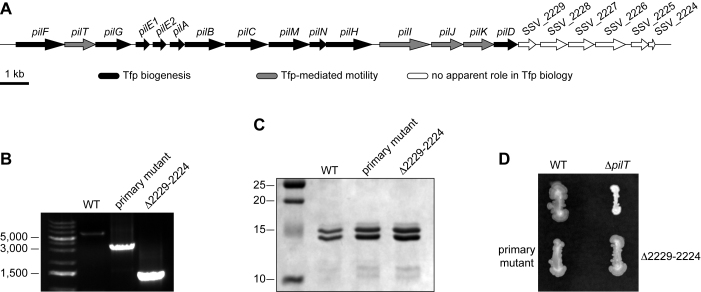
Creation of multiple gene deletion mutants. A markerless *Δ*2229-2224 deletion mutant shows that the last six genes in the *pil* locus are dispensable *en bloc* for Tfp biology. (**A**) Schematic representation of the *pil* locus in *S. sanguinis* 2908. Genes in white, which can be deleted individually with no obvious efects on Tfp biology (6), were deleted *en bloc*. (**B**) PCR analysis of a Km^S^ clone selected on *p*-Cl-Phe plates after transformation of a *Δ*2229-2224::pheS*aphA-3 primary mutant by a sPCR product in which the Up and Down regions flanking these six gene have been spliced together. WT and primary mutant have been included as controls. The expected PCR amplicons are 6,098 bp for the WT, 3,266 bp for the primary mutant, and 1,407 bp for markerless *Δ*2229-2224 deletion mutants. Sizes shown are in bp. (**C**) Analysis of piliation. Tfp purifed by shearing/ultra-centrifugation were separated by SDS-PAGE and stained with Coomassie blue. WT and primary mutant were included as controls. Samples were prepared from cultures adjusted to the same OD_600_, and identical volumes were loaded in each lane. (**D**) Analysis of motility. Bacteria were streaked on TH plates, incubated several days at 37°C in a humid atmosphere before the plates were photographed.

A 22 kb cluster named *pil* (Figure 4A), which encompasses 21 genes, encodes all the proteins involved in Tfp biology in 2908 (6). A systematic marked mutagenesis showed that the last six genes in this cluster could be individually deleted without measurable effect on piliation or twitching motility (6). To confirm that the above two-step methodology is broadly applicable for gene deletion in *S. sanguinis*, we used it to delete *en bloc* the last six genes in the *pil* locus (SSV_2229 to SSV_2224) and we determined whether this had any effect on Tfp biology.

After splicing together the double cassette with the Up region from SSV_2229 and Dn region from SSV_2224 (Figure 4A), and transforming the sPCR1 product directly into 2908, we created a primary mutant in which the last six genes in the *pil* locus were replaced by the pheS*aphA-3 double cassette. When this primary mutant was transformed with a sPCR2 product in which the Up and Dn regions were spliced together to generate an in-frame markerless deletion (Figure 1C), we found that half of the colonies growing on *p*-Cl-Phe plates indeed corresponded to markerless *Δ*2229-2224 deletion mutants (Table 2). This was confirmed by PCR (Figure 4B) and sequencing for a few clones. Therefore, our gene editing technology is broadly applicable in *S. sanguinis* for the creation of markerless deletions, which can encompass several genes. Next, we prepared filaments from the unmarked *Δ*2229-2224 mutant and analysed them by Coomassie staining after SDS-PAGE (Figure 4C), which showed that this mutant yielded pili indistinguishable from those of the WT strain (6). These pili were functional and could mediate twitching motility as revealed by the presence of spreading zones present around *Δ*2229-2224 mutant colonies grown on agar plates (Figure 4D). Together, these findings suggest that the last six genes in the *pil* locus do not play any detectable role in Tfp biology since they can be deleted *en bloc* without affecting Tfp biogenesis or Tfp-mediated motility.

### *In situ* creation of missense *pilD* mutants shows that *S. sanguinis* prepilin peptidase PilD contains two Asp residues essential for its enzymatic activity

**Figure 5.**
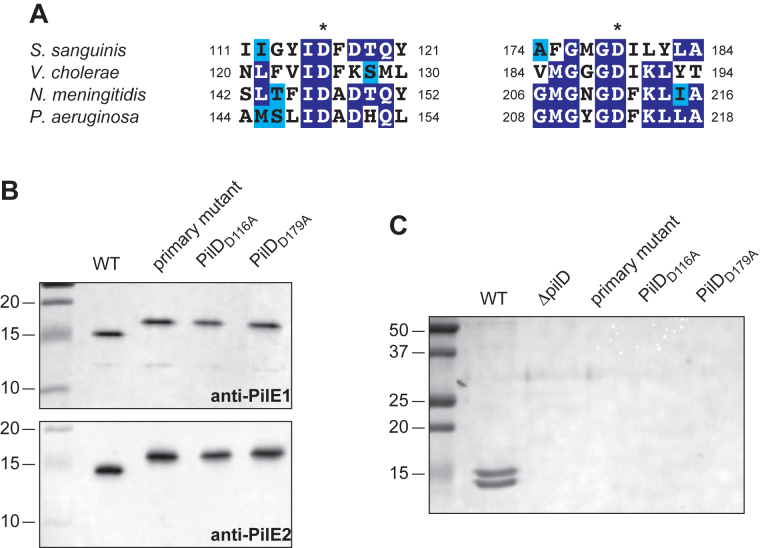
Creation of missense mutants. The prepilin peptidase PilD contains two Asp residues that are essential for its enzymatic activity. (**A**) Multiple sequence alignment of the regions in prepilin peptidases containing the two catalytic Asp residues (indicated by *) in *S. sanguinis, V. cholerae, N. meningitidis* and *P. aeruginosa*. Residues were shaded in dark blue (when identical), light blue (when conserved) or unshaded (when different). (**B**) Immunoblot analysis of PilD pilin processing activity. Whole-cell protein extracts from markerless PilD_D116A_ and PilD_D179A_ mutants in which missense mutations were introduced in the two catalytic Asp residues were probed using anti-PilE1 and anti-PilE2 antibodies. WT and primary mutant were included as controls. Protein extracts were quantified and equalised, and equivalent amounts of total proteins were loaded in each lane. (**C**) Analysis of piliation. Tfp purifed by shearing/ultra-centrifugation were separated by SDS-PAGE and stained with Coomassie blue. WT, marked *ΔpilD* and primary mutant were included as controls.

Tff pilin subunits are always synthesized as precursors that need to be cleaved by a dedicated prepilin peptidase, which removes a short leader peptide, prior to polymerisation into filaments (7). Prepilin peptidases are aspartic acid proteases with two highly conserved catalytic Asp residues (10). Sequence alignments show that these Asp residues are also conserved in *S. sanguinis* PilD (Figure 5A), which we previously demonstrated to be important for processing the 18 residue-long leader peptides in PilE1 and PilE2 (6). To confirm that (i) our two-step methodology can be extended to the design of site-directed mutants and (ii) Gram-positive prepilin peptidases are aspartic acid proteases, we targeted the conserved Asp_116_ and Asp_179_ residues in *S. sanguinis* PilD to determine whether they are essential for its enzymatic activity.

As above, using sPCR, we created a ΔpilD::pheS*aphA-3 primary mutant in which the *pilD* gene encoding *S. sanguinis* prepilin peptidase was replaced by our double cassette (Figure 1C). In parallel, we used site-directed mutagenesis on a cloned *pilD* gene to engineer two missense mutant alleles encoding PilD_D116A_ and PilD_D179A_ variants. When the primary mutant was transformed with these mutant alleles (Figure 1C), half of the clones growing on *p*-Cl-Phe plates had the desired mutant genotype (Table 2), which has been confirmed by sequencing for a few clones. Therefore, our gene editing technology in *S. sanguinis* can also be used for the creation of markerless missense mutations *in situ*.

To test the functionality of the PilD_D116A_ and PilD_D179A_ variants, we prepared whole-cell protein extracts and detected by immunoblotting the two major pilus subunits PilE1 and PilE2 using previously generated antisera (6). As can be seen in Figure 5B, in the PilD_D116A_ and PilD_D179A_ variants we detected proteins of higher molecular weight than the mature 14.7 and and 14 kDa pilins detected in the WT strain. The masses of the PilE1 and PilE2 proteins detected in the PilD_D116A_ and PilD_D179A_ mutants are consistent with unprocessed 16.9 and 16.2 kDa precursors, similarly to what is observed in a marked *ΔpilD::aphA-3* mutant in which the gene was deleted (Figure 5B). The Asp_116_ and Asp_179_ residues in *S. sanguinis* PilD are therefore essential for the ability of this enzyme to cleave pilin substrates. Consequently, the corresponding mutants were non-piliated since no pilin bands were present in their pilus preparations (Figure 5C). Together, these findings show that the two highly conserved Asp residues in prepilin peptidases are important for the enzymatic activity of Gram-positive enzymes.

### *In situ* engineering of the *pilE1* gene to encode a protein with a C-terminally appended 6His tag shows that the C-terminus of pilin subunits represents a permissive insertion site

One of the main limitations when studying a protein of interest is the necessity to generate specific antibodies, which is most often expensive and time-consuming. It was obvious to us that one of the major uses of our two-step methodology could be to engineer genes to append affinity tags to protein of interest, which could then be detected using commercial antibodies.

**Figure 6.**
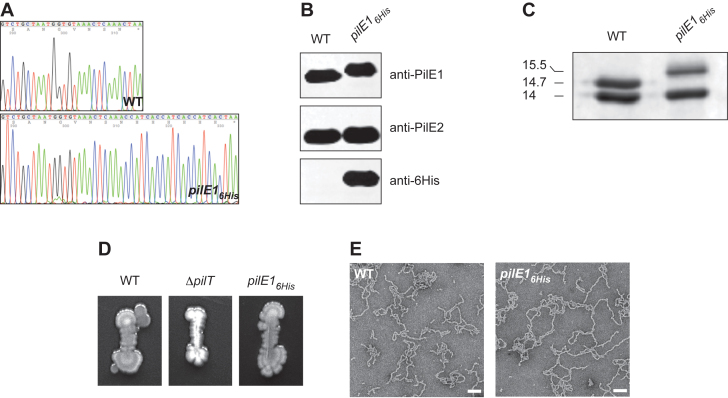
Engineering genes to encode proteins fused to an affinity tag. C-terminally 6His-tagged major pilin PilE1 can be detected using commercial antibodies and is assembled in functional filaments. (**A**) Trace sequence files of the C-terminus of the *pilE1* gene in WT and *pilE1_6His_*, which encodes a protein with a C-terminally fused 6His tag. (**B**) Immunoblot analysis of PilE1_6His_ expression. Whole-cell protein extracts were probed using anti-PilE1 or a commercial anti-6His antibody. They were also probed using anti-PilE2 antibody, as a control. Protein extracts were quantified and equalised, and equivalent amounts of total proteins were loaded in each lane. (**C**) Analysis of piliation. Tfp purifed by shearing/ultra-centrifugation were separated by SDS-PAGE and stained with Coomassie blue. WT was included as a control. (**D**) Analysis of motility. Bacteria were streaked on TH plates, incubated several days at 37°C in a humid atmosphere before the plates were photographed. (**E**) Analysis of WT and *pilE1_6His_* pilus preparations by TEM after negative staining. Scale bars represent 100 nm.

As above, using sPCR, we created a ΔpilE1::pheS*aphA-3 primary mutant in which the *pilE1* gene encoding one of the two pilin subunits in 2908 was replaced by our double cassette (Figure 1C). This primary mutant was then transformed with a sPCR2 product in which a sequence encoding a 6His tag was fused to the C-terminus of *pilE1*. As confirmed by PCR and sequencing (Figure 6A) for a few clones, many colonies growing on *p*-Cl-Phe plates indeed corresponded to strains in which the target gene was replaced by an allele encoding a PilE1_6His_ protein. To determine whether the tagged protein was expressed, we prepared whole-cell protein extracts and detected it by immunoblotting using anti-PilE1 or commercial anti-6His antibodies. As can be seen in Figure 6B, the tagged protein could be detected by both antibodies, while the PilE1 protein expressed by the WT strain could not be detected using the commercial anti-6His antibody. PilE1_6His_ displays a sligthly higher molecular weight than WT PilE1, consistent with the addition of the tag. Importantly, PilE1_6His_ was still capable of being assembled into filaments, as seen by SDS-PAGE/Coomassie on purified pilus preparations (Figure 6C). These filaments were functional since they could mediate twitching motility (Figure 6D). Moreover, they were morphologically indistinguishable from those purified from the WT strain as assessed by TEM (Figure 6E). Together, these results indicate that the C-terminus of PilE1 is permissive for insertion of epitopes, without interfering with this pilin's ability to be assembled into Tfp.

## DISCUSSION

Owing to its relative ease of manipulation and natural competence, *S. sanguinis* has become a Gram-positive workhorse for genomics, which culminated in the design of a comprehensive and ordered collections of mutants (5). Advanced genetic analysis such as construction of polymutants is hindered by the paucity of antibiotic resistance cassettes available, and no robust method is available for markerless modification of genes of interest *in situ*. In this report, we have addressed these limitations by designing a powerful method for directed and markerless engineering of *S. sanguinis* genes.

Our simple two-step method uses a mutant pheS* gene as a counterselectable marker (15). As previously shown in distant species belonging to Proteobacteria and Firmicutes (16–18), the PheS_A316G_ variant promotes sensitivity to the phenylalanine analog *p*-Cl-Phe in *S. sanguinis* and can be used for creating markerless deletion mutants rapidly and reliably. Since no antibiotic resistance cassette is present in these mutants, this method can be used iteratively for the creation of polymutants, which will allow the study of epistatic interactions between genes. Critically, although this was not explored in other species (16–18), it was obvious to us that this two-step method should allow any type of markerless gene editing, beyond the creation of markerless deletion mutants. We have therefore successfully used it to introduce specific point mutants in genes of interest and to engineer genes to encode proteins with appended affinity tags. Since these modifications are introduced *in situ*, the level of expression of the studied genes is not affected unlike in classical experiments when similarly modified genes are present on self-replicating plasmids or integrated ectopically, often under different promoters. Since PheS is universally conserved and a mutant PheS* promotes sensitivity to *p*-Cl-Phe even in the presence of the WT allele (the mutation is dominant), there is no need for a modified strain and this method could therefore be broadly used. Nevertheless, it seems particularly well adapted for species that can be transformed by PCR products, like naturally competent bacteria or model species where recombineering is possible (22). In these species, a cloning-independent approach can be used, which considerably reduces necessary time and effort compared to cloning-based approaches. This also circumvents toxicity/stability issues often associated with cloning of heterologous DNA in *E. coli*. The only caveat is that the current promoterless double cassette is limited to engineering genes whose promoter is strong enough to drive expression of pheS*aphA-3. For genes of poor/undetectable expression, it might be necessary to create a cassette in which pheS*aphA-3 expression would be driven by well-known constitutive or inducible promoters (23,24).

Tfp have only recently been discovered in Gram-positive species (25,26), including in *S. sanguinis* (6), while they have been studied for decades in Gram-negative species (8). The rudimentary nature of Tfp in Gram-positive bacteria (27) suggests that they might be better suited to tackle some outstanding questions. Our markerless gene engineering method further establish *S. sanguinis* as a Gram-positive model to study Tfp biology. Therefore, to illustrate the power and utility of our method we have focused on several genes in the *pil* locus, which contains all the genes dedicated to Tfp biology in this species (6). Firstly, despite the complex operonic structure of this target gene (Figure 2A), we confirmed that a markerless *ΔpilT* deletion mutant, which encodes the PilT pilus retraction motor (28), exhibits the same phenotype as a marked mutant (6). Besides validating our method, this finding paves the way for the creation of double mutants to determine whether the piliation defect in some *pil* genes could be alleviated in the absence of pilus retraction, as was described in model piliated Gram-negative species (29–32). Secondly, the rudimentary nature of the Tfp machinery in *S. sanguinis* has been further demonstrated by showing that the last six genes in the *pil* locus can be simultaneously deleted without adverse effects on piliation or Tfp-mediated motility. Hence, the first 15 genes in the *S. sanguinis pil* locus are sufficient for the biogenesis of retractable Tfp. It remains possible, however, that some of the deleted genes play a role in other Tfp-mediated functions that are yet to be discovered. Thirdly, we show for the first time that Gram-positive prepilin peptidases are also aspartate proteases with two aspartic acid residues essential for cleavage activity, as demonstrated earlier in a variety of archaea and Gram-negative bacteria (10,33–36). This is an important finding as it confirms the evolutionary relationship between distant Tff, suggesting that results pertaining to Tfp biology obtained in *S. sanguinis* will have general implications. Finally, by appending an affinity tag to one of the two major pilin subunits in *S. sanguinis*, we illustrated one of the most useful applications of our gene editing technology, *i.e*. modification of genes *in situ* to encode proteins with appended tags that can be easily detected using commercial antibodies. Importantly, we showed that the C-terminus of Gram-positive pilins is permissive for the insertion of foreign epitopes as previously seen with some Gram-negative pilins (37,38). This is perhaps unexpected considering the widely different C-terminus in Gram-positive pilins, where the almost universal stabilising disulfide bond found in pilins composing Gram-negative Tfp (39) is absent. This paves the way for affinity chromatography purification of 6His-tagged Tfp, which might be amenable to detailed structural study and should allow the respective roles of PilE1 and PilE2 to be precisely determined.

In conclusion, the broadly applicable method we describe here for sophisticated markerless *S. sanguinis* gene editing *in situ* is expected, in the years to come, to help us shed light on the biology of an important opportunistic pathogen and of filamentous nano-machines almost universal in prokaryotes.
